# Determination of morpho-physiological and yield traits of maize inbred lines (*Zea mays* L.) under optimal and drought stress conditions

**DOI:** 10.3389/fpls.2022.959203

**Published:** 2022-07-28

**Authors:** Maha G. Balbaa, Hassan T. Osman, Essam E. Kandil, Talha Javed, Sobhi F. Lamlom, Hayssam M. Ali, Hazem M. Kalaji, Jacek Wróbel, Arkadiusz Telesiñski, Adam Brysiewicz, Rehab Y. Ghareeb, Nader R. Abdelsalam, Ahmed M. Abdelghany

**Affiliations:** ^1^Maize Research Department, Field Crops Research Institute, Agriculture Research Center, Cairo, Egypt; ^2^Plant Production Department, Faculty of Agriculture (Saba Basha), Alexandria University, Alexandria, Egypt; ^3^College of Agriculture, Fujian Agriculture and Forestry University, Fuzhou, China; ^4^Botany and Microbiology Department, College of Science, King Saud University, Riyadh, Saudi Arabia; ^5^Department of Plant Physiology, Institute of Biology, Warsaw University of Life Sciences SGGW, Warsaw, Poland; ^6^Institute of Technology and Life Sciences-National Research Institute, Falenty, Poland; ^7^Department of Bioengineering, West Pomeranian University of Technology in Szczecin, Szczecin, Poland; ^8^Plant Protection and Biomolecular Diagnosis Department, Arid Lands Cultivation Research Institute, The City of Scientific Research and Technological Applications, Alexandria, Egypt; ^9^Agricultural Botany Department, Faculty of Agriculture (Saba Basha), Alexandria University, Alexandria, Egypt; ^10^Crop Science Department, Faculty of Agriculture, Damanhour University, Damanhour, Egypt

**Keywords:** maize, inbred lines, principal component analysis, drought tolerance index (DTI), morpho-physiological, yield traits

## Abstract

Globally, climate change could hinder future food security that concurrently implies the importance of investigating drought stress and genotype screening under stressed environments. Hence, the current study was performed to screen 45 diverse maize inbred lines for 18 studied traits comprising phenological, physiological, morphological, and yield characters under optimum and water stress conditions for two successive growing seasons (2018 and 2019). The results showed that growing seasons and water regimes significantly influenced (*p* < 0.01) most of the studied traits, while inbred lines had a significant effect (*p* < 0.01) on all of the studied traits. The findings also showed a significant increase in all studied characters under normal conditions compared to drought conditions, except chlorophyll content, transpiration rate, and proline content which exhibited higher levels under water stress conditions. Furthermore, the results of the principal component analysis indicated a notable distinction between the performance of the 45 maize inbred lines under normal and drought conditions. In terms of grain yield, the drought tolerance index (DTI) showed that Nub60 (1.56), followed by Nub32 (1.46), Nub66 (1.45), and GZ603 (1.44) were the highest drought-tolerant inbred lines, whereas Nub46 (0.38) was the lowest drought-tolerant inbred line. These drought-tolerant inbred lines were able to maintain a relatively high grain yield under normal and stress conditions, whereas those drought-sensitive inbred lines showed a decline in grain yield when exposed to drought conditions. The hierarchical clustering analysis based on DTI classified the forty-five maize inbred lines and eighteen measured traits into three column- and row-clusters, as inbred lines in cluster-3 followed by those in cluster-2 exhibited greater drought tolerance in most of the studied traits. Utilizing the multi-trait stability index (MTSI) criterion in this study identified nine inbred lines, including GZ603, as stable genotypes in terms of the eighteen studied traits across four environments. The findings of the current investigation motivate plant breeders to explore the genetic potential of the current maize germplasm, especially in water-stressed environments.

## Introduction

Maize (*Zea mays* L.) is one of the world’s most important crops for food security because it feeds millions of people (FAOSTAT, 2017; El-Naggar et al., 2020; Kandil et al., 2020). Maize production on a global scale totaled 1.40 billion Mg in 2018, spread across a 236 million hectares surface area (FAOSTAT, 2021). Maize is one of the most widely grown crops in developing countries, where it’s grown on 100 million hectares (Rosegrant et al., 2009; Gomaa et al., 2021; Abdelsalam et al., 2022). By 2050, demand for maize in developing countries is predicted to quadruple, while worldwide production is expected to peak in 2025, with the majority of that production coming from developing countries (Shiferaw et al., 2011). Nonetheless, maize yields have been severely constrained in a number of developing countries due to a variety of abiotic and biotic stresses, as well as other causes (Salika and Riffat, 2021). Drought, heat, and flooding are becoming more common, putting a burden on a number of vital crops, as a result of rising urbanization and habitat degradation as well as other unpredictable extreme climatic phenomena (Javed et al., 2020; Ahmad et al., 2021, 2022a; Chowdhury et al., 2021; Salika and Riffat, 2021; Shabbir et al., 2021; Akhtar et al., 2022). Maize crop development is also challenged in the long run by the intensifying global climate. According to climate change scenarios, agricultural productivity will be drastically reduced, limiting the ability of many regions to make the technological advances necessary for future food security (Cairns et al., 2012). Millions of impoverished maize consumers may face hunger and food insecurity unless farmers produce climate-resilient cultivars that boost yields (Lobell et al., 2008).

Drought is one of the most deleterious abiotic stresses in agricultural production, posing a threat to the global food supply (Gamalero and Glick, 2012; Vurukonda et al., 2016; Gupta et al., 2022; Singhal et al., 2022). If this condition continues, it is anticipated that 30% of the world’s water supplies will be depleted, and the number of regions experiencing drought will more than double by the year 2050 (Van Dijk et al., 2013; Williams et al., 2013; Van Loon et al., 2016). According to the available literature, arid and semi-arid regions would experience frequent hot droughts, which will have an impact on agricultural productivity (Yandigeri et al., 2012; Hessl et al., 2018). Considerably, drought can alter the characteristics of soil, restrict nutrient mobility, and inhibit microbial decomposition activities in soil (Rillig et al., 2019; De Vries et al., 2020). Under drought stress, plants have a decreased ability to efficiently absorb water and nutrients, show a reduced rate of photosynthesis and hormonal balance, in addition to having increased accumulation of reactive oxygen species (Arora et al., 2002).

The intensity of the drought, duration of the exposure, and growth stage influence maize yield loss (Kamali et al., 2022). It is impossible to forecast when drought stress will strike a plant because it can happen at any time of a plant’s life cycle. Despite this, the vast majority of research indicates that the blooming stage of the maize plant is the stage of growth that is most vulnerable to drought (Anjum et al., 2011). As a consequence, earlier breeding efforts have concentrated on drought resistance during the blooming and grain filling stages, with less an emphasis placed on seedlings’ ability to tolerate drought stress (Qayyum et al., 2012; Meeks et al., 2013). However, Qayyum et al. (2012) discovered that dryness at the seedling stage was just as significant as drought during the flowering stage. Particularly, if it happens when the seedlings are still young, drought stress can reduce crop stand (Khayatnezhad et al., 2010). Consequently, fewer plants typically attain their physiological maturity, which ultimately results in lower agricultural production. Meeks et al. (2013) suggested that screening maize cultivars when they are still in the seedling stage could assist in the identification of drought-tolerant inbred lines at upcoming growth stages. There have been relatively few investigations into the veracity of this hypothesis were carried out inside maize breeding programs (Djemel et al., 2018). Drought exposure at the seedling stage significantly reduces overall maize biomass, while drought exposure throughout the jointing to milk phases modifies the maize phenotypic and reduces yield (Zhu et al., 2021; Cao et al., 2022; Saad-Allah et al., 2022). In addition, the rate of photosynthesis is lower during the tasseling stage than it is during the jointing stage or the milking stage when the drought intensity is the same (Mi et al., 2018; Ahmad et al., 2022b,c). In addition, it was found that maize plants exposed to water stress during the seedling stage postponed in the dates of their anthesis and maturity, while simultaneously experiencing an extension of the development period (Song and Jin, 2020). Other studies found that water stress during the vegetative and tasseling phases caused a reduction in total biomass as well as an early loss of lower leaves. This led to a decreased grain production during the ear development and milking stages as a result of a lesser amount of solar energy being gathered (Cakir, 2004).

Due to the wide range of genetic variations among the various accessions of maize, it serves as a model organism for genetic research (Strable and Scanlon, 2009; Afzal et al., 2020; Ali et al., 2021; Bahar et al., 2021; Mahmood et al., 2021; Abbas et al., 2022). During the previous century, conventional breeding was highly effective at increasing the yield potential of crops, and this was largely accomplished with little knowledge of the factors controlling the genetic variability exploited by breeders, particularly for abiotic tolerance (Collins et al., 2008). Several recent and previous studies reported a bunch of genes governing tolerance to drought stress in maize (Strable and Scanlon, 2009; Mao et al., 2015; Fan et al., 2016; Wang et al., 2016; Xiang et al., 2017; Zenda et al., 2019). Maize ZmPYL gene expression profiles have previously been studied in response to ABA and dehydration stress (Fan et al., 2016). In addition, drought-tolerance genes ZmNAC111 and ZmVPP1 were recently discovered using a genome wide association study in a maize population comprising 368 accessions (Mao et al., 2015; Wang et al., 2016). A natural variation in ZmDREB2.7 was found to be significantly associated with drought tolerance (Liu et al., 2013b). Increased ZmVPP1 and ZmTIP1 gene expression improved root biomass and root hair elongation, implying a more developed root system that may contribute to maize drought resistance (Zhang et al., 2020).

Indeed, plants respond to drought stress in a variety of ways (Gaufichon et al., 2010; Islam et al., 2021; Ahamd et al., 2022a; Akhtar et al., 2022; Kumar et al., 2022; Shabbir et al., 2022). When plants are exposed to drought, they typically exhibit a series of morphophysiological changes associated with tolerance, including the maintenance of free proline accumulation, stomatal conductance, chlorophyll content, substomatal carbon dioxide concentration, canopy temperature, plant height, and stem diameter (Mafakheri et al., 2010; Ali et al., 2011; Araus et al., 2012; Gao et al., 2015). Among such adapting scenarios is proline accumulation (Kemble and Macpherson, 1954; Moussa and Abdel-Aziz, 2008; Špoljareviæ et al., 2011; Hayat et al., 2012). Kemble and Macpherson (1954) were the first to observe the accumulation of free proline in drought-stressed plant tissues. Other reports illustrated that maize cultivars were successfully screened for drought tolerance using the proline determination assay (Špoljareviæ et al., 2011; Abdelghany et al., 2021). Proline accumulation in drought-stressed plants enables surviving and recovery by preserving the structure of cell proteins, scavenging for hydroxyl radicals, and regulating cell reduction and oxidation reactions, among other roles (Hayat et al., 2012). Also, Moussa and Abdel-Aziz (2008) reported that accumulation of free proline has been utilized as a marker for drought tolerance, whereas other research stated that free proline has been used to screen for drought resistance in a variety of plant species (Liu et al., 2013a). Plants need to control leaf stomatal conductance to acquire CO_2_ and prevent desiccation (Dodd, 2003). Some drought-tolerant maize genotypes reduce stomatal conductance more during drought (Ray and Sinclair, 1997). Also, previous reports indicated that chlorophyll content decreases under drought stress, especially in drought-sensitive cultivars (Kuroda et al., 1990; Gholamin and Khayatnezhad, 2011).

Traditional breeding programs have indeed contributed to the development of high-yielding and drought-tolerant genotypes (Silva et al., 2020). Thus, the objectives of this study were: (a) to examine morphophysiological, biochemical responses, and yield characteristics of forty-five maize inbred lines grown under two different water constraint regimes, regular irrigation and water stress, (b) to identify the best-performing maize inbred lines under both normal and water-stressed conditions, and (c) determine the stability of forty-five maize inbred lines using multi-trait stability index (MTSI) criterion.

## Materials and methods

### Screening of maize parents

Two field experiments were conducted at a Farm in Nubaria Region, El- Behira Governorate, Egypt during 2018 and 2019 seasons, to evaluate 45 maize lines for growth, yield, and its components under two water regimes: normal and stress conditions to identify the tolerant inbred and sensitive inbred for water stress based on different measured traits.

The sources from which grains of 45 white maize lines were gained and pedigree are shown in Table 1. The grains were sown on May 20 and 18 of 2018 and 2019 seasons, respectively. The experiment was laid out in a split-split plot design with four replicates, whereas the two seasons were the main plot, meanwhile water stress (normal and stress) were occupied at sub-plots, while the 45 lines distributed in sub-sub plot. The two water regimes and their durations were presented in Table 2.

**TABLE 1:** The code number, names, pedigree, and origin of the 45 maize inbred lines.

No.	Name	Pedigree	Country of origin
1	Nub-1A	G2-E(S6)-5-1-1-1-1	ARC(Egypt)
2	Nub 5	G2-E(S6)-30-1-2-1-1	ARC(Egypt)
3	Nub 6	G2-E(S6)-32-1-2-2-2	ARC(Egypt)
4	Nub 8	G2-E(S6)-49-4-2-1-3	ARC(Egypt)
5	Nub 10	G2-E(S6)-60-2-2-1-2	ARC(Egypt)
6	Nub 11C	G2-E(S6)-69-4-1-1-2	ARC(Egypt)
7	Nub 11D	G2-E(S6)- 69-4-1-1-3	ARC(Egypt)
8	Nub 15	G2-E(S6)-156-1-1-1-2	ARC(Egypt)
9	Nub 22	G2-E(S6)-215	ARC(Egypt)
10	Nub 26A	G2-E(S6)-244-1-1-1-1	ARC(Egypt)
11	Nub 26B	G2-E(S6)- 244-1-1-1-2	ARC(Egypt)
12	Nub 26C	G2-E(S6)- 244-1-1-1-3	ARC(Egypt)
13	Nub 26D	G2-E(S6)-244-2-1-2-2	ARC(Egypt)
14	Nub 27	G2-E(S6)-262	ARC(Egypt)
15	Nub32	G2-E(S6)-290	ARC(Egypt)
16	Nub 34C	G2-E(S6)-320-3-1-3-3	ARC(Egypt)
17	Nub 35	G2-E(S6)-334-2-1-1-5	ARC(Egypt)
18	Nub 36	G2-E(S6)-335-1-1-2-3	ARC(Egypt)
19	Nub 37	G2-E(S6)-365-1-1-1-3	ARC(Egypt)
20	Nub 39	G2-E(S6)-472-1-1-2-1	ARC(Egypt)
21	Nub 45C	AED(S5)-16-5-1-1	ARC(Egypt)
22	Nub 46	AED(S5)-65-3-1-1	ARC(Egypt)
23	Nub 52	(G102*Sd63)-1	ARC(Egypt)
24	Nub 60	CIM.28-8	Cimmyt
25	Nub 66	Pop.38-2-1-1	ARC(Egypt)
26	Nub 74	Pop.38-13-2-2	ARC(Egypt)
27	Nub 80	Pop.38-143-1-1	ARC(Egypt)
28	Nub 84	(CML373)P43SR	Cimmyt
29	Nub 85	(CML442)TL10B-6903-139	Cimmyt
30	Nub 86	(CML445) TL10B-6903-140	Cimmyt
31	Nub 87	(CML483) TL07A-1903-237	Cimmyt
32	Nub 90	(CML538)HA09175-3	Cimmyt
33	Sd 7	A.E.D × an exotic composite, A4	ARC(Egypt)
34	Sd 17	G2-E-7DR	ARC(Egypt)
35	Sd 34	A. E. D.	ARC(Egypt)
36	Sd 63	Teplacinco # 5 (Tep-5)	Mexican
37	SK8	Population SK7	ARC(Egypt)
38	SK9	SC10 *SK43	ARC(Egypt)
39	SK12	Population SK14	ARC(Egypt)
40	SK13	SC sd1050*Gm30	ARC(Egypt)
41	GZ602	B73*Sd7	ARC(Egypt)
42	GZ603	B73HA*Sd7	ARC(Egypt)
43	GZ612	B73 (P-90 Bsss-1) x Sd7	ARC(Egypt)
44	GZ613	B73*Sd7	ARC(Egypt)
45	GZ628	B73 (P-90 Bsss-1) x Sd-62	ARC(Egypt)

**TABLE 2:** The irrigation treatments for maize crop during 2018 and 2019 seasons.

Cultural practices	Normal	Stress
1st irrigation	After 21days	After 21days
2nd irrigation	After 33 days	After 33 days
3rd irrigation	After 45 days	Escaped 45 days
4th irrigation	After 57 days	Escaped 57 days
5th irrigation	After 69 days	After 69 days
6th irrigation	After 81 days	After 81 days
7th irrigation	After 93 days	After 93 days

Each plot included one ridge/inbred (6 m in length and 0.70 m in width) with the distance between hills (25 cm). All agricultural practice were conducted according to the recommendation of Ministry of Agriculture and Land Reclamation for Nubaria Region. The preceding crop was wheat in the first and second seasons. Soil texture was sandy loam with high content of total CaCo_3_%.

#### Measuring of drought tolerance index

Drought tolerance index (DTI) was assumed according to (Fernandez, 1992) as follows:


$$\mathrm{DTI}=\left(Y_{\text{s}}\times Y_{\text{p}}\right)/\left({\bar{\text{Y}}}_{\text{p}}^{2}\right)$$

*DAS: Days after sowing.

Where Y_*s*_, Y_*p*_, and Y_*p*_ represent mean performances of studied trait under water stress conditions, under normal conditions for each genotype and overall mean under normal conditions for all genotypes, respectively. When STI is ≥1.0, it indicates that genotype is tolerant, If STI is <1, it indicates that genotype is sensitive.

### Data recorded

Data were collected for the following characteristics from samples taken from 10 random plants from two center ridges.

#### Phenological characteristics

1. *Days to 50% tasseling (DT, days)*: expressed as number of days from planting to the day when 50% of the plants had tassels in each sub-plot.
2. *Days to 50% silking (DS, days)*: expressed as the number of days from planting to the day when 50% of the plants are in silk emergence stage.

#### Physiological characteristics

The following agronomic characteristics were measured:

##### Leaf area (cm^2^)

The leaf area (LA) was determined according to the method described by Radford (Radford, 1967).

LA = K (L * W). Where LA = leaf area (cm^2^); K = Constant (0.75); L = leaf length (cm) and W = Maximum leaf width (cm).

##### Relative water content

To obtain an accurate measurement of relative water content (RWC), fully expanded younger leaves from each treatment were gathered. After the surface of the leaf had been carefully dried with tissue paper, it was first wrapped in polythene bags and then transported to the laboratory. To determine the fresh weight of the leaf, samples of the leaf were weighed (FW). After that, the samples were placed in plastic tubes that contained distilled water and allowed to sit in the dark for an entire night. The following morning, these leaves were delicately swollen with tissue paper to determine the turgid weight, and the results were recorded (TW). After that, a hot air oven was used to dry the leaves at a temperature of 70°C until the weight remained the same. After that, dried leaves were weighed to record their dry weight (DW). The RWC was determined by applying the formula presented below according to Schonfeld et al. (1988) as follow:


RWC(%)=(FW-DW)/(TW-DW)× 100

##### Transpiration rate (TR, mmol m^–2^ s^–1^) and stomatal conductance (SC, mol m^–2^ s^–1^)

An infrared gas analyzer (IRGA) Leaf Chamber Analyzer collected these two gas exchange properties from fully expanded flag leaves for each treatment during the anthesis stage (Type LCA-4, United States). A sunny day with a CO2 content of 0.05% was used for the measurements, which were taken from 10:00 a.m. to 11:59:59 a.m of 400 μmol mol^–1^ (Long and Bernacchi, 2003).

##### Chlorophyll content (SPAD)

The SPAD502 chlorophyll meter was used to test chlorophyll content at four developmental stages: anthesis, 14, 28, and 42 days post-anthesis (Minolta Co., Ltd., Osaka, Japan). The portable apparatus uses the absorbance of two light wavelengths (650 and 940 nm) flowing through intact leaves to estimate the amount of chlorophyll present. All plants were tested for chlorophyll content even if there were no competing plants in the immediate vicinity of the samples. The highest ear’s ear leaf was used to take the measurements. The base, middle, and midway between the midrib and the leaf border were all measured (Gekas et al., 2013). For each individual plant, the readings were repeated three times, and the average of those values was recorded.

##### Leaf proline content (PC, mg/g)

Proline content in fresh-leaf samples was determined as a physiological measure of the plant’s health in response to water stress treatments. There were three stages of grain filling observed during the sampling period of 80 days after planting (DAP). Sampling was done from 11:00 a.m. to 2:20 p.m. local time. Each plot included two plants, and we collected leaf disks from both. In the meantime, the leaf disk had been submerged in a chilled solution for proline extractions (3% aqueous sulfosalicylic acid solution). Before extracting and determining the leaf proline content, the samples were transported to a cold conditions and refrigerated (Bates et al., 1973). Samples were measured by spectrophotometer and repeated twice.

#### Morphological characters

A sample of ten random ears from each plot was used to determine the following traits:

1. Plant height (PH,cm): ten guarded plants from each entry were selected at maturity and plant height was measured with a meter rod in centimeters from the ground level to the base of the tassel and the average height was calculated.
2. Ear height (EH, cm): measured from the ground level to the upper bearing node of the same plants used in measuring plant height.
3. Ear length (EL, cm): length of the ear (cm) measured from 10 random ears/plot.
4. Ear diameter (ED, cm): measured as an average of the same 10 ears used in ear length estimation.
5. Cob diameter (CD, cm): measured as an average of the same 10 ears used in ear length estimation.

#### Yield and yield components

The yield and its components in this study were recorded as follows:

1. Number of rows/ear (NRE): Number of ears per plant were recorded before harvest at maturity stage, only ears containing 10 kernels or more were included in the count according to Zelleke (2000).
2. Number of kernel/row (NKR): The number of kernels per unit area was calculated from complete grain sample at maturity using a seed counter.
3. 100-kernel weight (HKW, g): taken randomly from grains of the same 10 ears after shelling (g) adjusted at 15.5% grain moisture. The 100-kernel weight was also determined from the same sample according to Tollenaar and Lee (2006).
4. Number of ear/plant (NEP): was measured by counting all ears of the ten randomly selected plants.
5. Grain yield (GY, ardbe/fed): was measured and adjusted to 15.5% grain moisture then converted to grain yield in ardbe/feddan (ardbe = 140 kg).

### Statistical analysis

Analysis of variance (ANOVA) at a significant level of *P* < 0.05 was performed with SAS 9.4 (SAS Institute Inc., Cary, NC, United States). Tukey’s test was also applied in SAS to compare the significance of the two water treatments at probability of *P* < 0.05, and further presented in boxplot which was constructed in the statistical software R (R Core Team, 2021) version 4.1.1 using *ggplot2* package. The DTI values were used to construct a two-way hierarchical clustering heatmap using the R package *ComplexHeatmap*. Radar chart was developed using Excel-Stat (Ahmed et al., 2022), displaying DTI values relative to a center point for the 18 examined traits. The R package *corrplot* was implemented to analyze correlation matrix plot, while the two R packages *FactoMineR* and *factoextra* were used to generate principal component analysis (PCA) biplot. The multi trait stability index (MTSI) with 20% selection intensity was analyzed using the *metan* R package (Olivoto and Lúcio, 2020).

## Results

### Performance of maize inbred lines in normal and drought conditions

**FIGURE 1:**
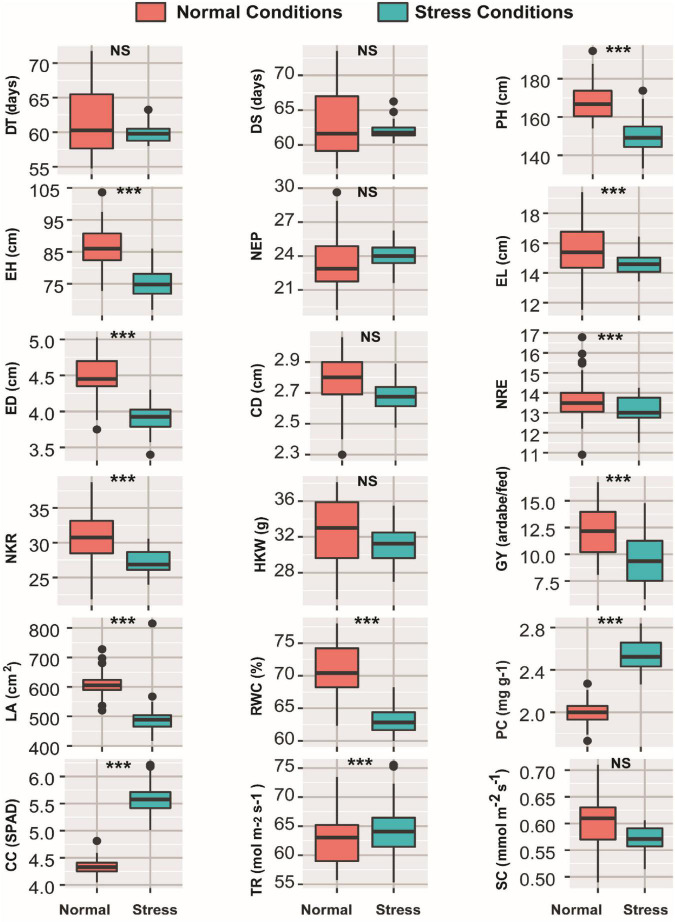
Boxplots showing variation in all 18 morpho-physiological and biochemical measured traits of 45 maize inbred lines grown under normal and drought conditions. *** and NS denote significant variation between treatments at 0.1% levels of probability and non-significant, respectively. DT, days to 50% tasseling; DS, days to 50% silking; PH, plant height (cm); EH, ear height (cm); NEP, no. of ears/plant; EL, ear length (cm); ED, ear diameter (cm); CD, cob diameter (cm); NRE, no. of row/ear; HKW, 100-kernel weight (g); NKR, number of kernel/row; GY, grain yield (ardbe/fed); LA, leaf area (cm^2^); RWC, relative water content (%); PC, proline content (mg g^– 1^); CC, chlorophyll content (SPAD); TR, transpiration rate (mmol m^– 2^ s^– 1^); SC, stomatal conductance (mol m^– 2^ s^– 1^).

The results of ANOVA presented in Supplementary Table 1 revealed the individual effect of each of the growing season, water regimes, and inbred lines factors, as well as their interactions, on the 18 investigated traits. The effect of growing season was significant for all traits except for CD, NRE, and CC, whereas the effect of water regimes was significant for all traits with the exception of DT, DS, and NEM. The inbred lines significantly affected all studied traits. The interaction between year and treatment had a highly significant effect on all traits studied, except for TR, for which the interaction was not significant. All traits were unaffected by the interaction between year and inbred lines except for PH, CC, TR, SC, and GY, which were significantly affected. Difference between normal and drought stress conditions in respect to 18 studied traits was shown in Figure 1. The findings presented in Figure 1 showed that all studied traits showed significant increase in normal conditions compared to drought conditions, except CC and TR, and PC which recorded higher levels under drought conditions (Figure 1).

### Principal component analysis

**FIGURE 2:**
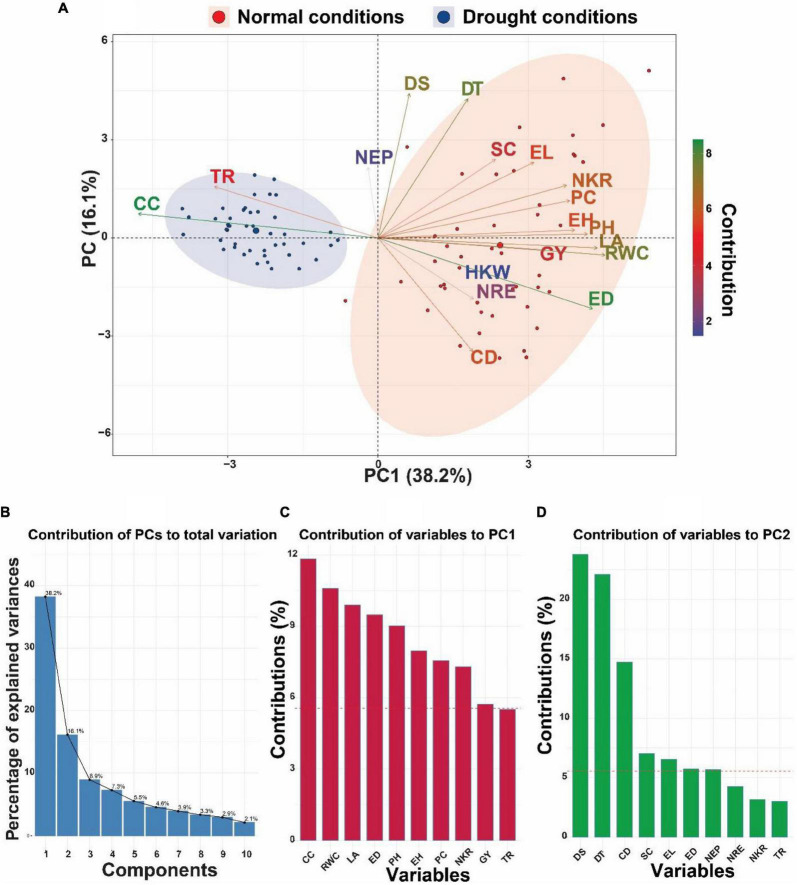
**(A)** Principal component analysis (PCA)-biplot of 45 maize inbred lines based on the variance in 18 morpho-physiological and biochemical traits grown under normal and drought conditions. Arrows indicate the strength of the trait influence on the first two PCs. The darker green and longer arrows indicate a higher contribution, while the darker blue and shorter arrows indicate the lower contribution of the variables. **(B)** Bar plots with% variation above represent contribution of each PC to the total variation. **(C,D)** Red dashed lines in the bar plots denote reference lines and the variable bars above the reference lines are considered most important in contributing to the PC1 and PC2. DT, days to 50% tasseling; DS, days to 50% silking; PH, plant height (cm); EH, ear height (cm); NEP, no. of ears/plant; EL, ear length (cm); ED, ear diameter (cm); CD, cob diameter (cm); NRE, no. of row/ear; HKW, 100-kernel weight (g); NKR, number of kernel/row; GY, grain yield (ardbe/fed); LA, leaf area (cm^2^); RWC, relative water content (%); PC, proline content (mg g^– 1^); CC, chlorophyll content (SPAD); TR, transpiration rate (mmol m^– 2^ s^– 1^); SC, stomatal conductance (mol m^– 2^ s^– 1^).

Principal component analysis (PCA) was performed on a dataset consisting of 45 maize inbred lines and 18 different variables to minimize the dimensionality of the data and show any potential correlations that may exist between the measured characteristics in this study (Figure 2). Under both drought and normal conditions, the associations that exist between the various factors and inbred lines, together with their respective major components, are displayed in biplot form (Figure 2A). The results of the biplot demonstrated that there was a discernible divide between the control group and the drought treatment (Figure 2A). As a result of the fact that the first two PCs accounted for the highest proportion of variance (54.3%, Figure 2B), the PCA-biplot was produced with the PC1 (28.2%) and PC2 (16.1%) (Figure 2A). The results of the biplot showed that characteristics such as TR and CC clustered together in the leftmost region of the biplot, scattering around the inbred lines under drought stress conditions.

Also, other traits such as DS, DT, SC, EL, NKR, PC, EH, PH, LA, RWC, GY HKW, ED, NRE, and CD were grouped in the rightmost part of the biplot concentrated close to the inbred lines under normal conditions, whereas NEP was positioned nearly in the center of the biplot, showing relatively similar performance under both water conditions. In respect to contribution of variables to the two PCs of the biplot (Figures 2B,C), the variable CC showed the highest contribution to PC1 (11.83%), followed by RWC (10.59%), LA (9.89%), ED (9.48%), and PH (9%) (Figure 2B), while contribution of variables to PC2 was largely explained by each of DS (238%) and DT (22.1%) (Figure 2C).

### Clustering of inbred lines and traits based on drought tolerant index

**FIGURE 3:**
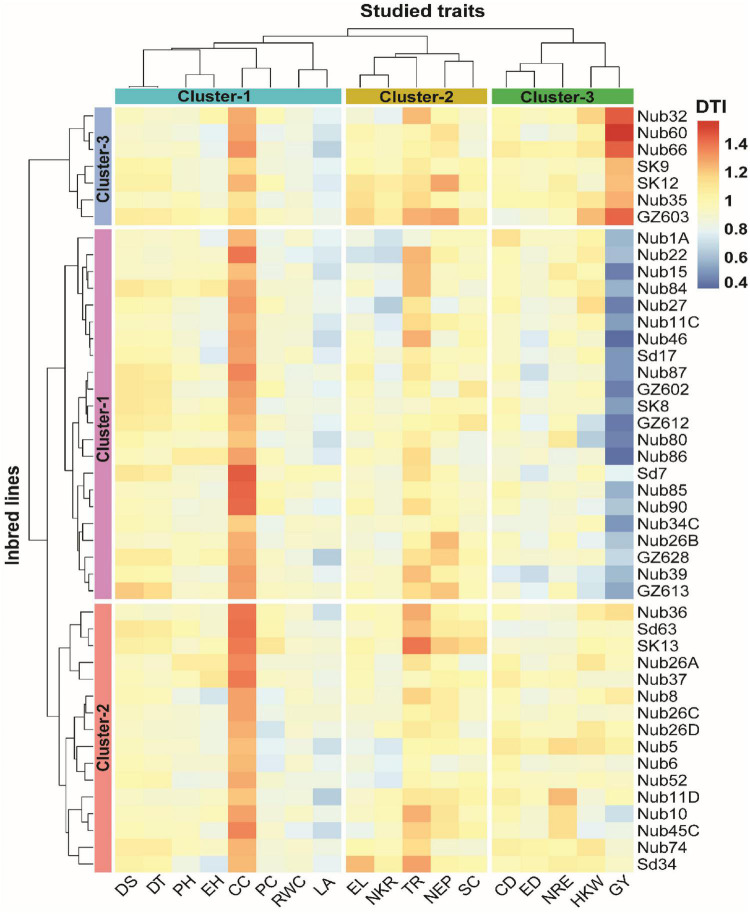
Hierarchical clustering and heatmap illustrating the associations among 45 maize inbred lines and 18 different traits in respect to drought tolerance index (DTI) condition. The different colors and intensities were adjusted based on cultivars–traits relationships. The darker blue-sky color indicates lower values (drought-sensitive), while the darker red indicates higher values (drought-tolerant). DT, days to 50% tasseling; DS, days to 50% silking; PH, plant height (cm); EH, ear height (cm); NEP, no. of ears/plant; EL, ear length (cm); ED, ear diameter (cm); CD, cob diameter (cm); NRE, no. of row/ear; HKW, 100-kernel weight (g); NKR, number of kernel/row; GY, grain yield (ardbe/fed); LA, leaf area (cm^2^); RWC, relative water content (%); PC, proline content (mg g^– 1^); CC, chlorophyll content (SPAD); TR, transpiration rate (mmol m^– 2^ s^– 1^); SC, stomatal conductance (mol m^– 2^ s^– 1^).

A hierarchical clustering heatmap showing interrelationship among 45 inbred lines and 18 traits in response to drought tolerant index (DTI) is presented in Figure 3. DTI expresses the response of each inbred line across the two water regimes (normal and drought conditions) in respect to each of the 18 traits. The DTI values for all 45 maize inbred lines according to their response for the 18 measured traits are shown in Supplementary Table 2. The higher the DTI value for inbred lines, the less negatively effect of drought stress on each trait, and vice versa. Based on the DTI values, the highest range of DTI values was exhibited by GY (1.18) with a maximum DTI value of 1.56 recorded by inbred line Nub60 and minimum value of 0.38 recorded by Nub46. In contrast, the lowest range of DTI values was recorded by RWC (0.20) with a maximum value of 0.98 recorded by Sd7 and minimum value of 0.78 recorded by Nub22. Remarkably, the highest minimum (1.12 for Nub35) and maximum (1.46 for sd7) values were recoded by CC.

The findings of the hierarchical clustering indicated the classification of the 18 measured traits and 45 inbred lines into three clusters based on the variation existed in DTI. Based on variation in DTI, the 45 maize inbred lines were assembled into three row-clusters, as each of cluster-1, cluster-2, and cluster-3 consisted of 22, 16, and 7 inbred lines, respectively, joining of most closely associated inbred lines within each cluster. Cluster-1 and cluster-3 were joined in the same main cluster as most closely related to each other. Also, the 18 studied traits were grouped into three column-clusters, where cluster-1, cluster-2, and cluster-3 comprised 8, 5, and 5 traits, respectively. Each column-cluster contained the most related traits such as DS, DT, PH, EH, CC, PC, RWC, and LA in cluster-1, EL, NKR, TR, NEP, and SC in Cluster 2, whereas CD, ED, NRE, HKW, and GY were assembled in cluster-3. Based on the DTI values, the inbred lines in row-cluster 3, followed by those joined in cluster-2 exhibited greater drought tolerance, as showing lower higher DTI values in most of studied traits. Inbred lines assembled in row-cluster-1 showed the highest drought tolerance in term of GY due to having high DTI values especially for the four inbred lines Nub60 (1.56), Nub32 (1.46), Nub66 (1.45), GZ603 (1.44). In contrast, inbred lines in row-cluster-2 showed lower DTI value in term of GY, such as Nub46 (0.38), Nub86 (0.40), GZ612 (0.42), and Nub15 (0.43), showing high drought sensitivity.

**FIGURE 4:**
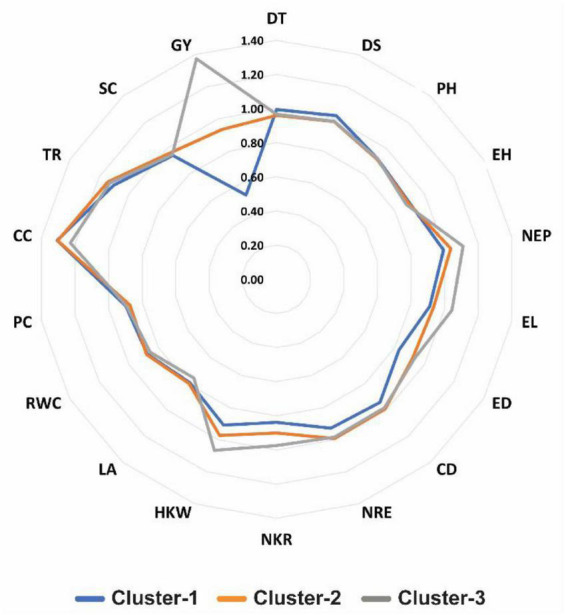
Radar plot showing drought tolerance index (DTI) values for 18 studied traits in three clusters of 45 maize inbred lines. Number in red color in the figure represent the complete scale of DTI. DT, days to 50% tasseling; DS, days to 50% silking; PH, plant height (cm); EH, ear height (cm); NEP, no. of ears/plant; EL, ear length (cm); ED, ear diameter (cm); CD, cob diameter (cm); NRE, no. of row/ear; HKW, 100-kernel weight (g); NKR, number of kernel/row; GY, grain yield (ardbe/fed); LA, leaf area (cm^2^); RWC, relative water content (%); PC, proline content (mg g^– 1^); CC, chlorophyll content (SPAD); TR, transpiration rate (mmol m^– 2^ s^– 1^); SC, stomatal conductance (mol m^– 2^ s^– 1^).

The findings of radar plot (Figure 4) indicated that the inbred lines assembled in cluster-3 exhibit high drought tolerance due to having high value of DTI for traits GY, NEP, EL, and HKW. Inbred lines in cluster-1 showed relatively high DTI values in term of DS, while CC was shown to be less effected by drought conditions, exhibiting low DTI values. Cluster-1 showed lowest drought tolerant inbred lines in many traits, including HKW, NKR, NRE, CD, ED, EL, NEP, and largely with GY. Besides, traits including PH, EH, PC, and RWC revealed reasonably similar response over all clusters (Figures 3, 4).

### Correlation analysis

**FIGURE 5:**
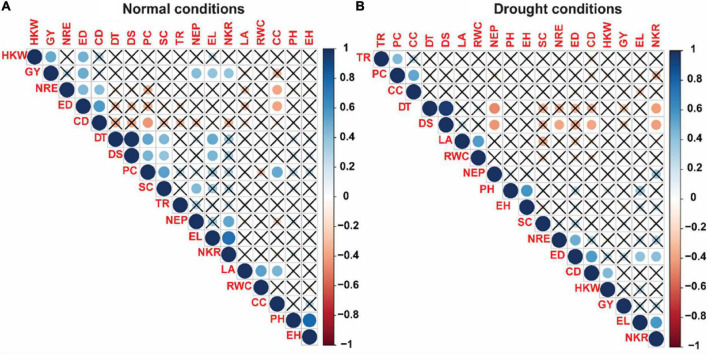
Correlation matrix of the 18 measured traits of 45 maize inbred lines evaluated under normal **(A)** and drought stress **(B)** conditions. The increasing color intensities illustrate a higher correlation coefficient. DT, days to 50% tasseling; DS, days to 50% silking; PH, plant height (cm); EH, ear height (cm); NEP, no. of ears/plant; EL, ear length (cm); ED, ear diameter (cm); CD, cob diameter (cm); NRE, no. of row/ear; HKW, 100-kernel weight (g); NKR, number of kernel/row; GY, grain yield (ardbe/fed); LA, leaf area (cm^2^); RWC, relative water content (%); PC, proline content (mg g^– 1^); CC, chlorophyll content (SPAD); TR, transpiration rate (mmol m^– 2^ s^– 1^); SC, stomatal conductance (mol m^– 2^ s^– 1^).

Pearson correlation coefficients among 18 studied traits in the current study were shown in Figure 5. Under control conditions (Figure 5A), phenological traits including DT and DS showed highly significant positive correlation with each other (0.99^***^), while both characters showed significant positive correlation with each of EL (0.51^**^, 0.46^**^), SC (0.44^**^, 0.39^**^), and EL (0.52^**^, 0.47^**^), respectively. For the physiological characteristics, a significant and positive correlation was observed between each pair of RWC and LA (0.53 ^**^), SC and PC (0.54^**^), CC and PC (0.51^**^), CC and LA (0.46), whereas significantly negative correlation was noticed between PC and CD (-0.43), and CC with each of NRE (-0.39^**^) and ED (-0.39^**^), while TR did show association with any of 18 studied traits. For morphological traits, most of traits showed significant and positive correlations such as between PH and EH (0.78^***^), EL and NKR (0.76^***^), EL and GY (0.39^**^), ED and HKW (0.48^**^), ED and GY (0.50^**^), ED and NRE (0.45^***^), CD and NRE (0.42^**^), CD and ED (0.57^**^), and CD and PC (-0.43^**^). For yield traits, GY showed highly significant correlation with each of and NKR (0.40^**^), NEP (0.43^**^), and HKW (0.51^**^).

As regard to correlation coefficients under drought conditions (Figure 5B), a significant positive correlation was shown between DT and DS (0.96^***^) which is similar with that was shown under control conditions. Also, DS correlated significantly and negatively with each of NEP (-0.44^**^) and NKR (-0.41^**^), NRE (-0.32*), and CD (-0.34^**^), whereas DT showed significant negative correlation with only NEP (-0.43^**^) and NKR (-0.41^**^). Regarding the physiological characteristics, significant positive correlation was revealed between each pair of TR and PC (0.43^**^), PC and CC (0.51^**^), and LA and RWC (0.56^**^), while correlation of physiological traits with other measured characters was observed non-significant. Interrelationshipamong morphological traits showed that significant and positive correlation was shown with each pair of PH and EH (0.58^***^), ED and CD (0.56^***^), and ED and EL (0.41^**^), while association of morphological traits with other studied characters showed significant and positive correlation only between ED and NKR (0.41^**^). For yield traits, no significant correlations were shown either among yield related traits or for yield related traits with other studied characters except a significant positive was shown between HKW and CD (0.45^**^).

### Multi-trait stability index and inbred lines selection

**FIGURE 6:**
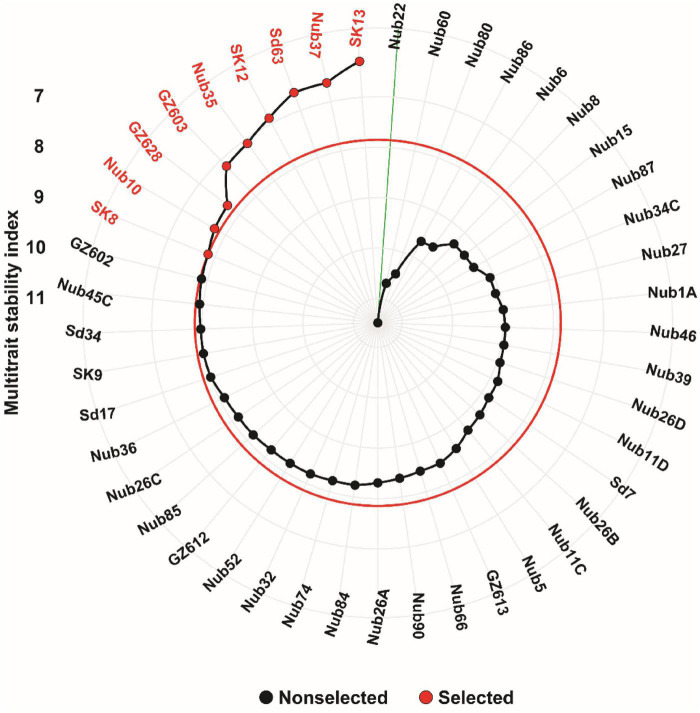
Ranking of 45 maize inbred lines based on MTSI values performed on 18 traits. The most stable inbred lines are shown in red color and the red circle represents the cut-point according to the selection intensity of 20%. The green line connected to the center of the plot represents the least stable inbred line with highest MTSI value.

The findings of MTSI performed for the 45 maize inbred lines across four environments (normal and drought conditions for two years) based on 18 studied traits are shown in Figure 6. Based on MTSI analysis, nine inbred lines representing a selection intensity of 20% of total 45 maize, including SK13, Nub37, Sd63, SK12, Nub35, GZ603, GZ628, Nub10, and SK8 were selected as the most stable inbred lines across the four environments. The MTSI values for all 45 maize inbred lines are shown in Supplementary Table 3. In terms of high stability and overall performance, these maize inbred lines represent the best materials out of the whole maize panel assessed. On the other hand, the most variable inbred lines were found in the inbred line Nub22, which recorded the highest value of MTSI (11.5).

## Discussion

The goal of maize breeders is to develop inbred lines of maize that are resistant to the effects of drought. Screening of a germplasm for its ability to withstand drought, particularly in settings in which the drought has been artificially induced, is an efficient method for choosing resources for advanced breeding programs. The fact that the effect of inbred lines was found to be extremely significant on all the measurable parameters that were recorded in this study suggests that the exploited germplasm represents a rich source of genetic variation that can be employed for advanced breeding programs. Therefore, the germplasm collection can be utilized to locate inbred lines that have high levels of tolerance to water stress (Adebayo et al., 2014). This can be determined by comparing the differential responses of the inbred lines under the two different water regimes. Since the inbred lines used in the study were selected from a variety of pedigrees and the majority of the recorded traits are quantitatively inherited, it was expected that the observed effects of the maize inbred lines and water treatments would be significant (Huqe et al., 2021). Evidently, the results of ANOVA showed highly significant variations between normal and drought stress conditions treatments for almost all the traits tested in this study. This suggests that there is a genetic difference between the maize cultivars that are used for drought tolerance (Chen et al., 2012). Morphological and growth characteristics, including PH, EH, EL, ED, and CD were significantly reduced under drought stress conditions. In contrast, physiological traits responded differently to drought stress conditions, where traits such as LA, RWC, and SC showed reduced values under drought conditions, while remaining traits PC, CC, and TR showed higher levels under drought conditions. The later physiological traits, namely PC, CC, and TR, played as key parameters introducing a differential response to drought stress, indicating good estimates of drought stress conditions, especially at the early vegetative stage of growth. The higher chlorophyll content, as well as transpiration under drought conditions in this study is similar with that previously reported (Sherin et al., 2022), which may ultimately cause an enhancement in photosynthetic rates. Furthermore, other studies also reported that under severe drought stress an increase in chlorophyll content was obtained and then, remained constant (Mensah et al., 2006). The results of this study also revealed that the higher levels of accumulated proline observed under drought conditions may illustrate an efficient mechanism for osmotic regulation and cellular adaptation to water stress which agrees with findings of earlier reports (Gunes et al., 2008). Such accumulation of proline could be utilized as an adaptive role for plants in drought-stressed areas (Solanki and Sarangi, 2014). On the other hand, the relative water content in current study showed a relative decrease for all genotypes under drought conditions which was in line with previous reports (Hussain et al., 2019). This also can be particularly noticed with inbred lines assembled in cluster-1, as they showed lower levels of RWC, providing one of the reasons for that decrease in grain yield under drought conditions for those cluster-1 inbred lines (Soltys-Kalina et al., 2016).

To enhance the screening efficiency of the 45 inbred lines under both optimal and drought stress conditions in this study, further discriminative analyses were performed, including hierarchical clustering analysis based on DTI to explore the nature of interrelationship between inbred lines and measured traits under diverse water conditions. According to the findings of respective DTI-based cluster analysis, the 45 inbred lines were grouped in three distinct cluster (cluster 1–3), each of which differed in number of inbred lines included. Cluster-2 and cluster-3, which were drought tolerant and moderately tolerant, respectively, showed higher and moderate levels of DTI, indicating lower and moderately lower loss in most of physiological and yield traits, especially GY, when exposed to drought stress conditions (Meeks et al., 2013; Maqbool et al., 2020). In contrast, cluster-1 showed the largest level of reduction in most of traits when exposed to water stress treatment. These findings are in accordance with those reported in a great number of previous studies (Hayat et al., 2012; Gupta et al., 2020; Huqe et al., 2021). Inbred lines can be maintained in high yielding ranks by selecting for higher grain production under both stressful and optimal conditions. This can be done through selective breeding (Cakir, 2004; Cairns et al., 2012). This is since identical inbred lines are going to be expected to perform admirably in either scenario. According to the finding of current study, it was revealed that certain inbred lines, such as Nub60, Nub32, Nub66, and GZ603 can preserve prominent high grain yields in both optimal and stressful conditions, which validates the findings that Foulkes obtained (Foulkes et al., 2007). Such tolerance response to drought stress exhibited by those elite inbred lines in this study could be mainly attributed to their genetic makeup, which controls the key traits in maize inbred lines under drought stress which is in accordance with several studies (Tuberosa, 2012; Min et al., 2016; Zenda et al., 2019).

In the current study, findings of Pearson correlation analysis among 18 studied traits showed an interesting correlation found between GY and yield traits such as NEP, HKW, and the NKR, which highlights the role of those yield components in contribution to high grain yield under normal conditions. However, there was no significant correlation between GY and its components with any of physiological traits including TR, PC, CC, and LA, and RWC under both conditions, explaining that it would be possible that grain yield may be mainly affected by its component rather that by variation in physiological parameters. Furthermore, under both control and drought conditions, physiological traits such as RWC showed significant positive correlation with LA, meaning that genotypes that maintained their water status may prevent membrane damage by improving antioxidant enzyme activity, which led to regulation of their photosynthesis activity under deficit water conditions. These findings thus revealed the prominence of these traits and drought indices in selecting tolerant genotypes for drought stress. The findings of the present study agree with that reported previously in other crops (Golabadi et al., 2006; Bahrami et al., 2014; Mohammadi, 2016; Ramakrishnan et al., 2016). Noticeably, a discrepant profile of association between studied traits under contrasting water regimes was exhibited in the current study. Collectively, significant negative and positive association found between various studied traits under water-deficit stress, further encourage employment of these associations for identifying promising maize drought-tolerant inbred lines. Otherwise, different patterns of correlations between same traits under varying water regimes should be considerably implemented since particular interrelations among some influential parameters under specific water conditions may act as selective criteria for genotypes with promising drought-responsive traits.

As another discriminative analysis applied on the data of the current investigation, PCA was utilized to find the most important selection characters for drought tolerance by using the first and second principal components. PCA–biplot is a technique of multivariate analysis in which traits and objects are combined in two dimensions, or more, whereas overlapping variations are minimized, making it easier to identify primary characters for selection (Arzu et al., 2018; Huqe et al., 2021). The PCA revealed that the variables, traits, CC, RWC, LA, ED, PH, DS, and DT played a larger role in characterizing variation between maize inbred lines. Findings of PCA also illustrated that variables TR and CC clustered together in the PCA biplot, closely scattering around the inbred lines under drought stress conditions, indicating their role as of great importance in selecting best characters under drought conditions. Results of PCA in this study also reveal that the yield and physiological variables have the potential to be utilized in selection for drought resistance. It can be concluded that drought-tolerant inbred lines can be identified using physiological traits as evidence from PCA. There are numerous studies that support these findings (Negrão et al., 2017; Zhao et al., 2019; Maqbool et al., 2020; Zhu et al., 2021; Alam et al., 2022).

Multi-trait stability index has recently been employed as a robust tool to assist in the selection of elite inbred lines based on the consistency and mean performance of various variables (Olivoto et al., 2019; Abdelghany et al., 2021; Olivoto et al., 2021; Zhu et al., 2021). Basically, the inbred lines that have lower values of MTSI suggest a higher level of stability based on the various measured attributes under study. In the current investigation, nine inbred lines were identified as highly stable inbred lines across four environments (two years and two water treatments) according to adopting a selection intensity of 20%. The selected stable inbred lines were SK13, Nub37, Sd63, SK12, Nub35, GZ603, GZ628, Nub10, and SK8, showing fair stability for all the 18 studied traits. Interestingly, this selection criterion was fairly justified under contrasting water environments for two years, illustrating the significance of the selection of inbred lines that perform best in terms of their consistency across optimum and stress water conditions. As a result, the selection of these inbred lines would be of tremendous assistance in enhancing the mean performance of the genotypes that were screened. In accordance with previous research on soybean, genotypes of soybeans that were resistant to drought and salinity were identified using the MTSI criterion (Zuffo et al., 2020; Youssef et al., 2021). Furthermore, an earlier study was carried to develop maize breeding procedures that are based on multivariate selection (Elmardy et al., 2021; Olivoto et al., 2021), and demonstrated that it is possible to discover hybrids that combine the desirable mean performance with stability for yield-related characteristics. Taken together, MTSI can enable breeders to discover hybrids that combine stress-adaptive features with the high yield, particularly under water stressed-environments.

## Conclusion

Here, we screened a panel of 45 maize inbred lines for 18 studied traits under two contrasting water regimes. Overall, most of the studies characters were significantly affected by seasons of study and water regimes, whereas variation due to inbred lines was significant for all studied traits. Our approach in this study pinpointed that testing diverse maize inbred lines under two contrasting water regimes resulted in remarkable change in the phenological, physiological, morphological, and yield traits. In contrast with other remaining traits, chlorophyll content, transpiration rate, and proline content showed high values under water stress conditions. Discriminative analyses used in this study such as PCA supported our methodology as a clear differential approach, indicating that chlorophyll content and transpiration rate traits were influential on performance of maize inbred lines under stress conditions, while other remaining traits were most discriminative under normal conditions. Furthermore, the use of drought tolerance index evaluated for all studied traits implied that Nub60, followed by Nub32, Nub66, and GZ603 were the highest drought-tolerant inbred lines, whereas Nub46 was the lowest drought-tolerant inbred line in response to grain yield. Utilizing MTSI approach in this study to examine their stability regarding multiple traits and multi environments of maize inbred lines led to identifying nine diverse inbred lines with high stability and prominent mean performance. Therefore, Nub60, Nub32, Nub66, and GZ603, as elite drought-tolerant lines identified in this study based on the drought tolerant index, could be recommended as promising parents for maize drought tolerance improvement breeding programs and as well as developing stable and high-performing lines. In addition, future studies can be conducted to investigate the molecular aspects of these promising inbred lines, such as investigating the molecular mechanism and expression profile of candidate drought-tolerant genes.

## Data availability statement

The original contributions presented in this study are included in the article/Supplementary material, further inquiries can be directed to the corresponding author.

## Author contributions

MB and NA: conceptualization. SL, HK, JW, AT, AB, RG, and AA: data curation. HO, EK, SL, RG, and AA: formal analysis. HK, JW, AT, AB: funding acquisition. MB, HO, HA, RG, and AA: investigation. MB, HO, and NA: methodology. HA, NA, HK, JW, AT, AB: project administration. EK, HA, and MB: resources. TJ, HK, JW, AT, AB, and SL: software. NA: supervision. HA, HK, JW, AT, AB, AA: visualization. NA, TJ, and MB: writing—original draft. TJ, NA, and AA: writing—review and editing. All authors contributed to the article and approved the submitted version.

## Supplementary material

Supplementary Table 1 Mean square values of measured traits obtained from two-way ANOVA (analysis of variance) in eighteen hybrid maize cultivars grown under control and salt stress environments.

Supplementary Table 2 The DTI values for all 45 maize inbred lines for the 18 measured traits.

Supplementary Table 3 MTSI values for all 45 maize inbred lines.
