# Erratum: Determination of morpho-physiological and yield traits of maize inbred lines (*Zea mays* L.) under optimal and drought stress conditions

**DOI:** 10.3389/fpls.2022.1069938

**Published:** 2022-11-15

**Authors:** Frontiers Production Office

**Affiliations:** Frontiers Media SA, Lausanne, Switzerland

**Keywords:** maize, inbred lines, principal component analysis, drought tolerance index (DTI), morpho-physiological, yield traits

Due to a production error, the affiliation linked to Adam Brysiewicz was incorrectly written as affiliation eight. This should be corrected to affiliation six. The publisher apologizes for this mistake.

The original version of this article has been updated.

