# Supplementary material for: Determination of morpho-physiological and yield traits of maize inbred lines (Zea mays L.) under optimal and drought stress conditions
Source: Front Plant Sci. 2022 Jul 28;13:959203. doi: 10.3389/fpls.2022.959203 (PMC9366912; doi:10.3389/fpls.2022.959203)
Supplement: Supplementary Table 1 — Mean square values of measured traits obtained from two-way ANOVA (analysis of variance) in eighteen hybrid maize cultivars grown under control and salt stress environments. [file Table_1.DOCX]

**Table S1**: Analysis of variance in fort-five maize inbred lines grown under control and drought stress conditions.

| **Traits** | **Year (Y)** | **Treatment (T)** | **Genotypes (G)** | **Y*T** | **Y*G** | **T*G** | **Y*T*G** |
| --- | --- | --- | --- | --- | --- | --- | --- |
| **Tass** | *** | NS | *** | *** | NS | *** | NS |
| **Silk** | *** | NS | *** | *** | NS | *** | NS |
| **PHT** | *** | *** | *** | *** | *** | NS | *** |
| **EHT** | *** | *** | *** | *** | NS | NS | NS |
| **EAR** | *** | NS | *** | *** | NS | *** | NS |
| **EL** | *** | *** | *** | *** | NS | *** | NS |
| **ED** | *** | *** | *** | *** | NS | *** | NS |
| **CD** | NS | *** | *** | ** | NS | *** | NS |
| **RPE** | NS | *** | *** | ** | NS | *** | NS |
| **KPE** | *** | *** | *** | *** | NS | *** | NS |
| **KWT** | *** | *** | *** | *** | NS | *** | NS |
| **LA** | *** | *** | *** | *** | NS | NS | NS |
| **RWC** | *** | *** | *** | *** | NS | NS | *** |
| **PC** | *** | *** | *** | *** | NS | NS | NS |
| **CC** | NS | *** | *** | *** | * | NS | NS |
| **TR** | *** | *** | *** | NS | *** | NS | *** |
| **SC** | *** | *** | *** | *** | *** | *** | *** |
| **GY** | *** | *** | *** | *** | *** | *** | *** |

*, **, and *** denote significant at 5%, 1% and 0.1% levels of probability, respectively. NS: Non-significant
